# Translucent Zone Between Autograft and Endplate Two Months Postoperatively Is an Independent Predictor of Delayed Osseous Union in Elderly Patients With Posterior Lumbar Interbody Fusion Surgery

**DOI:** 10.7759/cureus.30799

**Published:** 2022-10-28

**Authors:** Hiroki Ushirozako, Tomohiko Hasegawa, Shigeto Ebata, Tetsuro Ohba, Hiroki Oba, Keijiro Mukaiyama, Toshiyuki Ojima, Jun Takahashi, Hirotaka Haro, Yukihiro Matsuyama

**Affiliations:** 1 Department of Orthopedic Surgery, Hamamatsu University School of Medicine, Hamamatsu, JPN; 2 Department of Orthopedics, International University of Health and Welfare, Chiba, JPN; 3 Department of Orthopedic Surgery, University of Yamanashi, Chuo, JPN; 4 Department of Orthopedic Surgery, Shinshu University School of Medicine, Matsumoto, JPN; 5 Department of Orthopedic Surgery, North Alps Medical Center Azumi Hospital, Kitaazumi, JPN; 6 Department of Community Health and Preventive Medicine, Hamamatsu University School of Medicine, Hamamatsu, JPN

**Keywords:** elderly patient, teriparatide, posterior lumbar interbody fusion, delayed osseous union, cartilage endplate

## Abstract

Background

Delayed union or pseudoarthrosis after posterior lumbar interbody fusion (PLIF) is associated with poor outcomes in health-related quality of life. Therefore, it is important to achieve earlier solid fusion for a successful clinical outcome after PLIF. A few authors reported that biomechanical factors may influence spinal fusion rates. The purpose of our retrospective study was to evaluate the independent predictors of delayed osseous union related to intraoperative procedures of PLIF, and to find ways to reduce delayed osseous union.

Methods

This was a retrospective study of a completed trial. We reviewed 66 elderly patients with osteoporosis after PLIF (all female, mean age 71 years, follow-up period over 6 months). Lumbar computed tomography scans at 2 months postoperatively were examined for the presence of a translucent zone between autograft and endplate (more than 50% of vertebral diameter), and autograft position with bone bridging (anterior, central, or posterior). Osseous union was assessed by using computed tomography 6 months postoperatively.

Results

Thirty-three patients (50%) showed complete osseous union, while 33 did not. A translucent zone between autograft and endplate two months postoperatively was observed in nine patients (27%) in the union group and in 23 (70%) in the nonunion group (p<0.01). Autograft position with bone bridging two months postoperatively was anterior, central, and posterior in 17 (52%), 30 (91%), and 20 patients (61%) in the union group, and in 12 (36%), 20 (61%), and seven patients (21%) in the nonunion group (p=0.22, p<0.01, and p<0.01), respectively. Multivariate logistic regression analysis showed that the presence of a translucent zone between autograft and endplate (odds ratio, 0.101; 95% confidence interval: 0.026-0.398; p<0.01) and teriparatide administration (odds ratio, 8.810; 95% confidence interval: 2.222-34.936; p<0.01) were independently associated with osseous union after PLIF.

Conclusions

A translucent zone between autograft and endplate at two months postoperatively independently predicted delayed osseous union within six months after PLIF. Complete osseous union rates were higher in patients with posterior bone bridging two months postoperatively than in those without. These findings apart from preoperative predictors of osseous union might serve as indicators of how intraoperative techniques affects osseous union enhancement.

## Introduction

The posterior lumbar interbody fusion (PLIF) technique is widely used to treat various lumbar spine pathologies, including degenerative diseases, spondylolisthesis, foraminal stenosis, and adult spinal deformity, and to stabilize the affected motion segments [1,2]. However, postoperative complications related to poor bone quality, such as pseudoarthrosis and instrumentation failure, can occur [3,4]. Earlier solid fusion is important for a successful clinical outcome after PLIF.

Numerous factors, such as age, sex, osteoporosis, smoking, diabetes mellitus, hypertension, revision surgery, and medical comorbidities, are known to have significant impacts on the osseous union in spine surgery [5,6]. Many authors have reported the relationships between spinal fusion rates and patient characteristics or operative methods [7], and a few have reported that anatomical and biomechanical factors, such as the number of fused vertebral levels, cigarette smoking, and bone graft compositions, may influence spinal fusion success rates [8,9].

In our previous reports, it was demonstrated that weekly teriparatide administration and a preoperative anterior slippage < 2 mm of the cranial vertebra next to the fusion segment promoted intervertebral bone formation after PLIF [10,11]. As there are few reports on the relationship between the fusion rates and postoperative radiographic factors after PLIF, we analyzed these factors in this study. Therefore, we aimed to retrospectively evaluate the independent predictors of delayed osseous union related to intraoperative procedures of PLIF. We hypothesized that multiple biomechanical or radiographic factors would predict delayed osseous union.

## Materials and methods

Institutional review board approval

Our institutional review boards approved this multicenter randomized controlled trial, and it was registered with the University Hospital Medical Information Network (UMIN) clinical trials registry (UMIN000007151).

Participants

We conducted a retrospective analysis of the data obtained in the aforementioned randomized trial [10]. Seventy-five patients underwent single-level PLIF or transforaminal lumbar interbody fusion for degenerative spondylolisthesis, degenerative scoliosis, lumbar spinal stenosis, or isthmic spondylolisthesis at three university hospitals and their affiliated hospitals. Patients were randomly allocated to one of two groups: weekly treatment with teriparatide (56.5 µg), administered subcutaneously starting at one week postoperatively for six months, or no teriparatide. The inclusion criteria were 1) aged ≥50 years with lumbar degenerative disease; 2) female; 3) had a previous osteoporotic fracture and/or a bone mineral density (BMD) <80% of the sex-matched young-adult mean; 4) patients without histories of radiation treatment of the lumbar spine, high serum alkaline phosphatase or calcium levels, Paget bone disease, bone tumors, hypersensitivity to teriparatide, metabolic bone disease, or previous back surgery; and, 5) provision of informed consent for study participation. The exclusion criteria were patients with an adverse event or consent withdrawal, and a follow-up period <6 months. Postoperatively, all patients wore a soft corset for about three months and underwent physical therapy, including muscle strength training and walking. Most patients had complete data sets that were sufficient for analysis.

Surgical procedure of interbody space

In all patients, local autografts of adequate quantity were obtained bilaterally from the spinous processes, lamina, and facets. Discectomy and denudation of the cartilage endplates were performed to prepare the graft bed, and morselized local bone chips were packed in the anterior and lateral positions of the intervertebral space. One or two interbody cages filled with morselized local bone chips were inserted into the intervertebral space.

Radiographic, osteoporotic evaluation, and grouping

**Figure 1:**
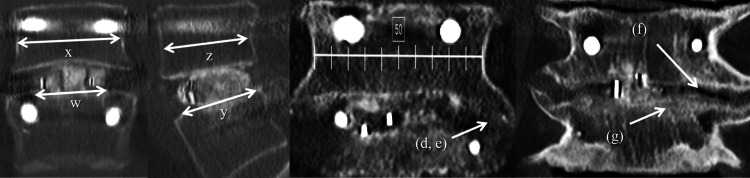
Definition of radiographic parameters using three-dimensional computed tomography scans (a) occupancy rate of autograft in coronal center slice = w/x; (b) occupancy rate of autograft in sagittal center slice = y/z; (c) total occupancy rate of autograft (occupancy rate of autograft in coronal and sagittal center slices) = a×b; (d) osteophyte formation (presence of anterior or lateral osteophytes ≥20% of vertebral diameter); (e) osteophyte union (presence of anterior or lateral osteophyte union); (f) presence of air in the intervertebral disc; (g) presence of osteosclerotic endplate.

**Figure 2:**
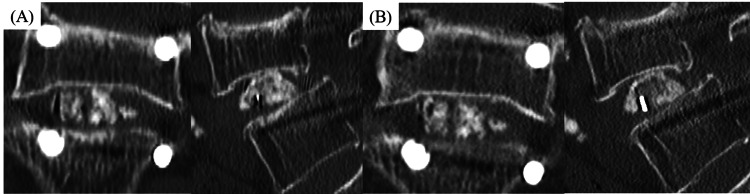
Representative case 1 (A) Case 1 with translucent zone comprising more than 50% of vertebral diameter between autograft and endplate two months postoperatively. Coronal CT slice in the center cage region was evaluated as grade II, sagittal CT slice in the central cage region was evaluated as grade I (for a total score of 3). (B) Case 1 without complete osseous union six months postoperatively. Coronal CT slice in the central cage region was evaluated as grade III, sagittal CT slice in the central cage region was evaluated as grade II (for a total score of 5).

**Figure 3:**
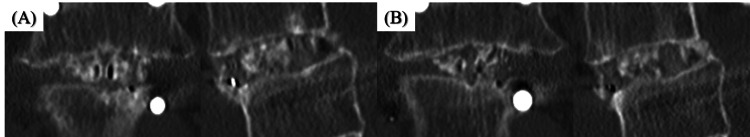
Representative case 2 (A) Case 2 with anterior, central, and posterior bone bridging at two months postoperatively. Both CT slices in the center cage region were evaluated as grade I, and each was given a score of 1 (for a total score of 2). (B) Case 2 with complete osseous union at six months postoperatively. Both CT slices in the center cage region were evaluated as grade I, and each was given a score of 1 (for a total score of 2).

The following radiographic parameters related to the intraoperative procedure of PLIF (Figure 1-3) were assessed by two orthopedic surgeons depending on each intervertebral fusion level: occupancy rate of autograft in coronal or sagittal center slices, total occupancy rate of autograft (occupancy rate of autograft in coronal and sagittal center slices), osteophyte formation (presence of anterior or lateral osteophytes ≥20% of vertebral diameter), osteophyte union (presence of anterior or lateral osteophyte union), presence of air in the intervertebral disc, presence of osteosclerotic endplate, presence of translucent zone between autograft and endplate (more than 50% of vertebral diameter), and autograft position with bone bridging (right, left, anterior, central, or posterior). The radiographic evaluations were similar to those in our previous report [11]. On flexion-extension radiographs, the angular motion was used to evaluate lumbar instability. At six months postoperatively, four doctors (blinded to the groups) independently evaluated intervertebral osseous union with dynamic radiography and three-dimensional computed tomography (CT) images, using the fusion grading system reported by Bridwell et al [12]. Bone construction was graded into three categories: grade I (1 point), which represents complete bony bridging with both superior and inferior vertebral body; grade II (as 2 points), which represents bony bridging with either superior or inferior vertebral body; and grade III (as 3 points), which represents incomplete bony bridging. Osseous fusion was assessed by two CT slices--the cage's center slices in both coronal and sagittal views. We defined complete intervertebral osseous union (bone fusion score of 2) as the presence of superior and inferior complete fusions in both coronal and sagittal CT slices (Figure 3B). A case with incomplete intervertebral osseous union was given a bone fusion score of 3 to 6 (Figure 2B). Procollagen type I amino-terminal propeptide and tartrate-resistant acid phosphatase 5b serum concentrations were evaluated as indicators of bone production and resorption, respectively, from samples taken preoperatively under non-fasting conditions [13]. Preoperatively, dual X-ray absorptiometry was used to assess femoral neck BMD. Patients were divided into two groups: those who successfully obtained complete intervertebral osseous union (bone fusion score = 2) and those who did not (non-union group).

Assessment of health-related quality of life (HRQOL)

Clinical and neurological symptoms were assessed preoperatively and every month postoperatively for six months using the Japanese Orthopaedic Association Back Pain Evaluation Questionnaire (JOABPEQ) [14,15], composed of parameters (pain-related disorders, lumbar spine dysfunction, gait disturbance, social life dysfunction, and psychological disorders) scored from 0 to 100 points (higher scores indicating better conditions), and the Oswestry Disability Index (ODI) [16]. Quality of life (QOL) scores for the five categories in the JOABPEQ were calculated for each patient preoperatively and six months postoperatively. According to the user’s guide for the JOABPEQ [14], an increase of 20 points or more between the preoperative and six-months postoperative scores indicated “effective” treatment.

Statistical analysis

The statistical analyses were similar to those in our previous report [11]. Categorical variables are expressed as absolute numbers and percentages and were analyzed by chi-square test or Fisher’s exact test as appropriate. We used Shapiro-Wilk tests to determine normality of distribution of continuous variables. Continuous variables with normal distributions, expressed as means ± standard deviations, were analyzed with unpaired t-tests. The amount of vertebral slippage, which was not normally distributed, is expressed as median and interquartile range (IQR) and was analyzed with the Mann-Whitney U-test. Predictors of osseous union after PLIF were examined with multivariate logistic regression analysis. Statistical analyses were conducted with SPSS version 23.0 (IBM, Armonk, NY, USA). P values ˂ 0.05 were considered significant.

## Results

This study included 75 patients, 37 with teriparatide and 38 without teriparatide. Nine patients (one consent withdrawal and eight adverse events) were excluded from this study. Finally, 66 patients were included in the analysis (mean age 71.0 years; all women; Table 1).

**Table 1:** Baseline, surgical, and osteoporotic data Values expressed as mean ± SD or number (percentage).

Variable	All (n = 66)
Age (years)	71.0 ± 7.4
Height (cm)	149.4 ± 6.5
Body weight (kg)	51.8 ± 8.1
Body mass index	23.2 ± 3.5
Hypertension	23 (34.8%)
Diabetes mellitus	7 (10.6%)
Rheumatoid arthritis	3 (4.5%)
Pretreatment of osteoporosis	5 (7.6%)
Teriparatide administration	29 (43.9%)
Condition	
Degenerative spondylolisthesis	53 (80.3%)
Degenerative scoliosis	1 (1.5%)
Lumbar spinal canal stenosis	18 (27.3%)
Isthmic spondylolisthesis	2 (3.0%)
Surgical treatment	
Posterior lumbar interbody fusion	62 (93.9%)
Transforaminal lumbar interbody fusion	4 (6.1%)
Level	
L3-L4	4 (6.1%)
L4-L5	56 (84.8%)
L5-S1	6 (9.1%)
Femoral bone mineral density (% young-adult mean)	
Femoral neck	74.0 ± 9.4
Total proximal	77.5 ± 11.8
Femoral bone mineral density (T-score)	
Femoral neck	-2.25 ± 0.83
Total proximal	-1.88 ± 1.01
Serum type I procollagen N-terminal propeptide (µg/L)	44.6 ± 18.9
Serum tartrate-resistant acid phosphatase 5b (mU/dL)	450.8 ± 180.3

Thirty-three patients (50%; union group) showed complete union (bone fusion score = 2) after PLIF, and 33 patients (non-union group) did not. Preoperative baseline, surgical, and osteoporotic data were not significantly different between both groups. Teriparatide was administered in 20 patients (60.6%) postoperatively in the union group and in nine patients (27.3%) in the non-union group (P = 0.006; Table 2). The radiographic data of both groups are shown in Table 2. At two months postoperatively, a translucent zone between the autograft and endplate was observed in nine patients (27.3%) in the union group and in 23 patients (69.7%) in the non-union group (P =0.001). At six months postoperatively, the total occupancy rate of autograft had decreased by 2.7% (from 56.1% to 53.4%) in the union group and 8.0% (from 53.1% to 44.9%) in the non-union group (P = 0.085). Of the patients treated with teriparatide, a translucent zone between the autograft and endplate was observed in six patients (30.0%) in the union group and in nine patients (100.0%) in the non-union group (P = 0.000) at two months postoperatively. The autograft position with bone bridging data in both groups is shown in Table 3. At two months postoperatively, autograft with bone bridging was positioned left, center, and posterior in in 15 (45.5%), 30 (90.9%), and 20 (60.6%) patients in the union group, and 7 (21.2%), 20 (60.6%), and 7 (21.2%) patients in the non-union group (P = 0.037, P = 0.004, and P = 0.001), respectively. After controlling for relevant confounding variables including age, body mass index, preoperative femoral neck BMD, and total occupancy rate of autograft, teriparatide administration (odds ratio [OR], 8.810; 95% confidence interval [CI]: 2.222-34.936; P = 0.002) and the presence of a translucent zone between autograft and endplate (OR, 0.101; 95% CI: 0.026-0.398; P = 0.001) were independently associated with osseous union within six months after PLIF (Table 4).

**Table 2:** Comparison of osteoporosis treatment and radiographic parameters in fusion segment between the union and nonunion groups Values expressed as mean ± SD or number (percentage). *statistically significant difference

Variable	Union group n = 33	Nonunion group n = 33	P-value
Teriparatide administration	20 (60.6%)	9 (27.3%)	0.006^*^
Two months postoperatively			
Number of cages	1.1 ± 0.3	1.1 ± 0.3	1.000
Occupancy rate of autograft in coronal slice (%)	66.7 ± 14.1	64.9 ± 16.2	0.628
Occupancy rate of autograft in sagittal slice (%)	83.8 ± 14.5	81.8 ± 11.5	0.544
Total occupancy rate of autograft (%)	56.1 ± 16.1	53.1 ± 15.5	0.453
Lateral osteophyte formation	19 (57.6%)	13 (39.4%)	0.139
Lateral osteophyte union	5 (15.2%)	1 (3.0%)	0.098
Anterior osteophyte formation	3 (9.1%)	0 (0%)	0.119
Anterior osteophyte union	0 (0%)	1 (3.0%)	0.500
Air in the intervertebral disc	10 (30.3%)	16 (48.5%)	0.131
Osteosclerotic change of lower endplate	8 (24.2%)	11 (33.3%)	0.415
Osteosclerotic change of upper endplate	10 (30.3%)	13 (39.4%)	0.438
Translucent zone between autograft and endplate	9 (27.3%)	23 (69.7%)	0.001^*^
Change amount (6 months – 2 months postoperatively)			
Occupancy rate of autograft in coronal slice (%)	-1.4 ± 6.5	-4.1 ± 11.5	0.241
Occupancy rate of autograft in sagittal slice (%)	-2.7 ± 10.9	-7.4 ± 14.4	0.140
Total occupancy rate of autograft (%)	-2.7 ± 9.9	-8.2 ± 15.2	0.085
Variable (Teriparatide group)	Union group n = 20	Nonunion group n = 9	P-value
Two months postoperatively			
Translucent zone between autograft and endplate	6 (30.0%)	9 (100%)	0.000^*^
Variable (Control group)	Union group n = 13	Nonunion group n = 24	P-value
Two months postoperatively			
Translucent zone between autograft and endplate	3 (23.1%)	14 (58.3%)	0.082

**Table 3:** Comparison of autograft position with bone bridging between the union and nonunion groups Values expressed as number (percentage). *statistically significant difference

Variable	Union group n = 33	Nonunion group n = 33	P-value
Two months postoperatively			
Right	12 (36.4%)	8 (24.2%)	0.284
Left	15 (45.5%)	7 (21.2%)	0.037^*^
Anterior	17 (51.5%)	12 (36.4%)	0.215
Central	30 (90.9%)	20 (60.6%)	0.004^*^
Posterior	20 (60.6%)	7 (21.2%)	0.001^*^

**Table 4:** Multivariate logistic regression analysis of adjusted risk factors related to osseous union after PLIF *statistically significant difference PLIF = posterior lumbar interbody fusion.

Variable	Odds ratio	P-value	95% confidence interval
Teriparatide administration (yes/no)	8.810	0.002^*^	2.222-34.936
Translucent zone between autograft and endplate (yes/no)	0.101	0.001^*^	0.026-0.398

Change in JOABPEQ and ODI scores

The change in JOABPEQ and ODI scores between the two groups is shown in Table 5. The preoperative scores did not differ significantly between the groups, and both scores improved significantly postoperatively in both groups. Neither scores were significantly different at six months postoperatively between the union and nonunion groups.

**Table 5:** Pre- and post-operative Japanese Orthopaedic Association Back Pain Evaluation Questionnaire and Oswestry disability index scores, and the changes (postoperative score - preoperative score) Values expressed as mean ± SD, or median (interquartile range). JOABPEQ = Japanese Orthopaedic Association Back Pain Evaluation Questionnaire; Group E = patients judged as “effective”; Group NE = patients not judged “effective”.

Variable	Union group n = 33	Nonunion group n = 33	P-value
Preoperative score in JOABPEQ			
Pain-related disorders	29 (14–71)	29 (0–43)	0.548
Lumbar spine dysfunction	42 (33–72)	50 (25–77)	0.810
Gait disturbance	25 (9–43)	21 (7–43)	0.585
Social life dysfunction	46 (30–51)	38 (27–51)	0.587
Psychological disorders	42 (29–48)	39 (30–50)	0.681
Change in the score (6 month postoperatively)			
Pain-related disorders	29 (29–71)	57 (0–57)	0.882
Lumbar spine dysfunction	17 (0–33)	17 (-25–33)	0.261
Gait disturbance	36 (21–57)	43 (21–57)	0.570
Social life dysfunction	22 (5–32)	19 (5–35)	0.822
Psychological disorders	18 (0–24)	10 (3–27)	0.549
The ratio of effective cases in JOABPEQ			
Pain-related disorders (Group E:NE)	22:3	21:5	0.703
Lumbar spine dysfunction (Group E:NE)	13:14	13:15	1.000
Gait disturbance (Group E:NE)	23:6	23:6	1.000
Social life dysfunction (Group E:NE)	15:14	13:15	0.793
Psychological disorders (Group E:NE)	13:17	9:20	0.422
Oswestry disability index			
Preoperative score	45.6 ± 14.0	45.6 ± 17.0	1.000
Change in the score (6 months postoperatively)	-19.5 ± 22.8	-20.8 ± 21.3	0.814

## Discussion

Our results showed that weekly teriparatide administration accelerated the fusion rate after PLIF. The presence of a translucent zone between autograft and endplate two months postoperatively was an independent predictor of delayed osseous union within six months after PLIF. Moreover, none of the patients with both translucent zone between autograft and endplate and teriparatide administration showed complete osseous union. Complete osseous union rates were higher in patients with posterior bone bridging two months postoperatively than in those without. These findings might serve as indicators of how intraoperative procedures affects osseous union enhancement. To attain successful bone fusion after PLIF, we believe that surgeons should perform complete discectomy, denudation of the cartilage endplates, and compact packing of the autograft, especially in the posterior disc space, as well as administer teriparatide weekly.

In serial post-contrast magnetic resonance imaging studies, Rajasekaran et al. reported that subchondral bone has rich vascularity, but cartilage endplates and discs have poor vascularity [17]. We evaluated the influence of intraoperative procedures on the fusion rate, given that the surgical techniques of cage insertion and bone grafting may affect fusion rate. In the current study, there was a significantly lower proportion of cases with a translucent zone between autograft and endplate observed on CT scans taken two months postoperatively in the patients with complete union than in those without. Moreover, complete osseous union rates were higher in patients with posterior bone bridging two months postoperatively than in those without. Unlike the risk factors of age and anatomical characteristics, avoidance of the introduction of a translucent zone or identifying the autograft position can easily be achieved during surgery. We believe that the translucent zone between autograft and endplate is related to the intraoperative remaining of the cartilage endplates or disc nuclear material; therefore, it is essential to remove the cartilage endplates with poor vascularity and pack the autograft behind the cage with the expectation of rich vascularity for osseous union enhancement after PLIF.

A previous preclinical study demonstrated that teriparatide administration enhanced lumbar spinal fusion, improved the quantity of fusion calluses, and accelerated spinal fusion [18]. Several clinical studies have shown that teriparatide administration promoted osseous union in patients with lumbar arthrodesis surgery [19-21]. Our results were in accordance with these findings; teriparatide administration was an independent predictor of intervertebral osseous union. Though the autograft absorption occurred between two and six months postoperatively, patients with complete osseous union had a suppression of the autograft absorption. Conversely, none of the patients with both a translucent zone between autograft and endplate and teriparatide administration showed complete osseous union. As the presence of a translucent zone between autograft and endplate is a big obstacle to osseous union, preparation of an autograft base bed intraoperatively is very important to accomplish complete osseous union.

Makino et al. reported that delayed union had a negative impact on the postoperative QOL outcomes using JOABPEQ score at the six-month follow-up [22]. In contrast, during short-term follow-up, there were no appreciable changes in pain and HRQOL scores between the osseous union and pseudoarthrosis groups after single-level spinal fusion surgery [23,24]. These subjects had lumbar spinal stenosis and degenerative spondylolisthesis, and involved only single-level arthrodesis surgery. In this study, ODI and JOABPEQ scores at six months postoperatively did not significantly differ between union and non-union groups. Our results supported the findings of previous studies, however, appreciable HRQOL changes might have not been observed given the short-term follow-up.

This study had a few limitations. First, it was a retrospective study with a six-month follow-up. Second, we evaluated intervertebral osseous union using CT images. The most definitive imaging method for assessing osseous union is CT, although this procedure exposes patients to radiation, which can pose a risk [25]. However, we employed CT methods that only exposed patients to 50% of the typical radiation dose. Finally, a few authors have reported that sagittal imbalance or mismatch of spinopelvic alignment may induce pseudoarthrosis after spinal arthrodesis [6,8,11]; however, because of the lack of whole-spine standing radiographs, we could not evaluate the effect of mechanical factors due to spinal alignment on delayed osseous union.

## Conclusions

We found that the presence of a translucent zone between autograft and endplate two months postoperatively was an independent factor of delayed osseous union within six months of PLIF. Furthermore, complete osseous union rates were higher in patients with posterior bone bridging two months postoperatively than in those without. These findings apart from preoperative predictors of osseous union after PLIF might serve as indicators of how intraoperative techniques affects osseous union enhancement.
